# Analysis of TDP-43 and its binding partners in neurodegenerative diseases

**DOI:** 10.1186/1750-1326-8-S1-P23

**Published:** 2013-09-13

**Authors:** Wejdan Kattuah, Claire Troakes, Tibor Hortobagyi, Boris Rogelj, Christopher Shaw

**Affiliations:** 1 Department of Clinical Neuroscience, Institute of Psychiatry, King’s College London, London, UK; 2 Department of Neuropathology, Institute of Pathology, Medical and Health Science Centre, University of Debrecen., Debrecen, Hungary; 3 Department of Biotechnology B3, Jozef Stefan Institute, Jamova 39, Ljubljana, Slovenia

## Background

Transactive DNA binding protein (TDP-43) is the major component of the ubiquitin-positive protein aggregates seen in ~90% of Amyotrophic Lateral Sclerosis (ALS) and 60% of Frontotemporal Lobar Degeneration (FTLD) cases [1]. We have previously shown that mutations in the gene encoding TDP-43 are causally linked to familial ALS+/-FTLD [2] TDP-43 belongs to the heterogeneous nuclear ribonucleoprotein (hnRNP) family of proteins that are involved in the regulation of RNA transcription, splicing, transport and translation [3]. There are a large number of hnRNPs, many of which have overlapping functions and often act cooperatively in RNA processing. Here we sought to determine whether TDP-43 aggregates contain other hnRNPs that might contribute to the neurodegenerative process.

## Materials and methods

Immunohistochemistry for 14 hnRNPs predicted to associate with TDP-43 were examined in brain and spinal cord tissues from 20 FTLD-TDP and ALS cases. Co-localization with TDP-43 was examined using double-labelling immunoflouresence. Expression of selected hnRNPs was then examined in other neurodegenerative cases and controls to determine the specificity of any changes observed.

## Results

One hnRNP demonstrated a striking accumulation within dystrophic neurites and cytoplasmic inclusions in the frontal cortex of FTLD-TDP cases. The hnRNP inclusions were not detected in other neurodegenerative cases with mutations of *MAPT*, *FUS*, *SOD1* and *C9ORF72.* This particular hnRNP was found to co-localize with ~85% of TDP-43 inclusions and ~67% of ubiquitin incluions, largely in the frontal cortex and hippocampus of FTLD-TDP cases. Interestingly, the inclusions were not seen in FTLD-TDP cases with C9ORF72 mutation.

## Conclusions

We have identified one hnRNP that is abundant within the inclusions seen in FTLD-TDP cases. The hnRNP is seen to colocalize with TDP-43 in the majority of cytoplasmic inclusions in FTLD-TDP but not other neurodegenerative disorders. The mechanistic implications of this interaction with TDP-43 and the contribution of its sequestration into inclusions towards neurodegeneration requires further investigation.

## References

[B1] Neumann M Sampathu D Kwong L Truax A Micsenyi M Chou T Bruce J Schuck T Grossman M Clark C McCluskey L Miller B Masliah E Mackenzie I Feldman H Kretzschmar H Trojanowski J Ubiquitinated TDP-43 in Frontotemporal Lobar Degeneration and Amyotrophic Lateral Sclerosis Science 2006 10 130 133 17023659 10.1126/science.1134108

[B2] Sreedharan J Blair IP Tripathi VB Hu X Vance C Rogelj B Ackerley S Durnall JC Williams KL Buratti E Baralle F de Belleroche J Mitchell JD Leigh PN Al-Chalabi A Miller CC Nicholson G Shaw CE TDP-43 Mutations in Familial and Sporadic ALS Science 2008 319 1668 1672 10.1126/science.1154584 18309045 PMC7116650

[B3] Buratti E Baralle FE Multiple roles of TDP-43 in gene expression, splicing regulation, and human disease Front Biosci 2008 13 867 878 10.2741/2727 17981595

